# Widespread reduction in sun-induced fluorescence from the Amazon during the 2015/2016 El Niño

**DOI:** 10.1098/rstb.2017.0408

**Published:** 2018-10-08

**Authors:** Gerbrand Koren, Erik van Schaik, Alessandro C. Araújo, K. Folkert Boersma, Antje Gärtner, Lars Killaars, Maurits L. Kooreman, Bart Kruijt, Ingrid T. van der Laan-Luijkx, Celso von Randow, Naomi E. Smith, Wouter Peters

**Affiliations:** 1 Wageningen University and Research, Wageningen, The Netherlands; 2 Embrapa Amazônia Oriental CPATU, Belem, Brazil; 3 Royal Netherlands Meteorological Institute (KNMI), De Bilt, The Netherlands; 4 University of Groningen, Centre for Isotope Research, Groningen, The Netherlands; 5 Instituto Nacional de Pesquisas Espaciais, São José dos Campos, Brazil

**Keywords:** Amazon rainforest, drought response, tropical terrestrial carbon cycle, El Niño-Southern Oscillation, sun-induced fluorescence, gross primary production

## Abstract

The tropical carbon balance dominates year-to-year variations in the CO_2_ exchange with the atmosphere through photosynthesis, respiration and fires. Because of its high correlation with gross primary productivity (GPP), observations of sun-induced fluorescence (SIF) are of great interest. We developed a new remotely sensed SIF product with improved signal-to-noise in the tropics, and use it here to quantify the impact of the 2015/2016 El Niño Amazon drought. We find that SIF was strongly suppressed over areas with anomalously high temperatures and decreased levels of water in the soil. SIF went below its climatological range starting from the end of the 2015 dry season (October) and returned to normal levels by February 2016 when atmospheric conditions returned to normal, but well before the end of anomalously low precipitation that persisted through June 2016. Impacts were not uniform across the Amazon basin, with the eastern part experiencing much larger (10–15%) SIF reductions than the western part of the basin (2–5%). We estimate the integrated loss of GPP relative to eight previous years to be 0.34–0.48 PgC in the three-month period October–November–December 2015.

This article is part of a discussion meeting issue ‘The impact of the 2015/2016 El Niño on the terrestrial tropical carbon cycle: patterns, mechanisms and implications’.

## 1. Introduction

Variations in the annual atmospheric increase of CO_2_ in the atmosphere (the so-called growth rate of CO_2_) are dominated by carbon exchange in the tropical regions [1–5]. Measurements of ^13^C in CO_2_ in the atmosphere show unequivocally that the terrestrial biosphere is the main driver of such variability [6]. The CO_2_ growth rate variations, in turn, correlate strongly with tropical temperature and precipitation anomalies [7]. Years with higher than average temperatures and lower than average precipitation over tropical land areas have led to the highest annual increases of atmospheric CO_2_ on record. This was used by Cox *et al.* [8] to calculate a climate sensitivity for tropical net ecosystem exchange (NEE), which can tentatively inform us on climate impacts over longer time scales, if the controlling mechanisms turn out to be the same.

Droughts play a central role in this mechanism, and the peak CO_2_ growth rates of 1983, 1997/1998, 2005, 2010 and 2015/2016 can all be traced back to the impact of excessive heat or lack of precipitation in the tropics. The effect of the 2010 drought on the Amazon net carbon balance was quantified using inverse modelling of vertical profiles of atmospheric CO_2_ and CO collected from aircraft over the Amazon forest [9–11]. All three studies found that during the dry year 2010, the Amazon rainforest was near neutral in its net CO_2_ exchange with the atmosphere in contrast with its functioning as a net carbon sink in 2011. Increased fires contributed substantially (0.1–0.3 PgC) to the anomalous annual CO_2_ budget, but reduced biospheric uptake (approx. 0.25 PgC) also played a significant role. An analysis by Bowman *et al.* [12] based on an inverse analysis of remotely sensed data agreed with the increased fire emissions, but suggested that equal increases in gross primary productivity (GPP) and respiration during 2010 left net ecosystem productivity unchanged.

An opportunity to study the effect of droughts on GPP is presented by sun-induced fluorescence (SIF), which is the re-emission of light by the chloroplast during photosynthesis. SIF can be retrieved from space-based remote sensing instruments aboard, for example, SCIAMACHY [13,14], MetOp [14–17], GOSAT [13,18–20], OCO-2 [21] and Sentinel-5P [22]. A fraction of the light detected by satellite instruments at the top-of-atmosphere around 740 nm originates directly from photosynthesis within vegetation foliage at the surface, and therefore is one of the most direct observations of primary productivity. Many studies have recently demonstrated the similarity between spatio-temporal patterns of SIF and of GPP [17,19,23–25], but there are just a few studies of SIF during tropical droughts.

In their 2013 study of the Amazon basin, Lee *et al.* [26] describe the seasonal cycle of SIF as retrieved from GOSAT and link it to the seasonal cycle of precipitation and vapour pressure deficits as observed during the 2010 drought. The strongest response comes from vegetation in the eastern part of the basin, which experiences seasonal droughts (precipitation less than 100 mm per month). SIF reductions over evergreen rainforests in the western part of the basin were difficult to distinguish in the short time series (January 2009–December 2010). Integrated over the full basin, SIF reduced by close to 15% in 2010 relative to its 2009 values though, suggesting a large impact of the 2010 drought on GPP. Bowman *et al.* [12] used GOSAT SIF in an inverse system to constrain GPP separately from NEE and respiration and suggested a reduction of GPP during the 2010 drought of 0.31 ± 0.20 PgC relative to 2011. Alden *et al.* [11] supported this finding and attempted to link qualitatively SIF to the inversely derived seasonal cycle of net carbon uptake by vegetation.

More recently, Liu *et al.* [5] also used GOSAT SIF in one of the first quantifications of the 2015/2016 El Niño impact on the tropical carbon balance. These authors came to the conclusion that the atmospheric CO_2_ increase was at least partially driven by a suppression of GPP in the Amazon region, but also by an increase of respiration over tropical Africa and an increase of tropical biomass burning over tropical Asia (also see Nechita-Banda *et al.* [27]).

Finally, Yang *et al.* [28] report a reduction of 8.2% in NASA-retrieved GOME-2 SIF [15] during the 2015/2016 drought for the Amazon region. This coincided with an overall greening up of the Amazon region, which these authors tentatively ascribe to increased light availability. The GOME-2 retrieval product used in their study is not particularly developed for high water vapour environments [29] and is subject to very high noise in the tropics, which translates into the lack of any clear spatial patterns in SIF over the Amazon in their analyses. Also, the degrading signal of GOME-2 SIF over recent years was not accounted for in this analysis, probably leading to incorrect conclusions on the 2015/2016 El Niño induced anomaly [30]. In a recent analysis of the drought response of tropical vegetation [31], this degradation played less of a role as many years of NASA-retrieved GOME-2 SIF were averaged to find a lack of GPP/SIF variations in response to precipitation variations in tall tropical trees with deep roots in the wettest part of the Amazon basin.

Here, we present an analysis of the impact of the 2015/2016 El Niño on sun-induced fluorescence from an update to the retrieval product called SIFTER [17]. Full details of this updated product (called SIFTER v2) are described by van Schaik [29], while our main focus here is a first analysis of the response of fluorescence to drought over the Amazon forest. SIFTER v2 is retrieved from GOME-2A, but compared to the product used in Yang *et al.* [28] it has the advantage of specifically accounting for substantial water vapour absorption signatures imprinted in the satellite spectra over the hot and humid Amazonian atmosphere. Relative to GOSAT SIF, the SIFTER product has a larger spatial footprint (80 × 40 km^2^ over most of the record) but achieves global coverage within 1 day, leading to many more valid retrievals over the cloudy tropical regions. Finally, GOME-2A spectra are available from 2007 onwards, providing us with a much longer background period (2007–2014) to contrast anomalies to than those obtainable from GOME-2B, OCO-2 or GOSAT. This allows us to very sharply define the observed seasonal cycles of SIF over the Amazon, as well as their anomalies during the recent El Niño.

## 2. Methods

### (a) SIFTER fluorescence

The level 3 (i.e. geospatial gridded data) SIFTER dataset has a temporal coverage from 2007 to 2017 for GOME-2A and from 2013 to 2017 for GOME-2B at a daily time resolution. It should be noted that the quality of the GOME-2A data decreases over time due to sensor degradation, while in June 2015 a change was made by ESA to the GOME-2B level-0 data that, for SIFTER, translates into less reliable retrievals past that moment. In this work, we use GOME-2A radiances and a slightly updated version of the SIFTER v2 retrieval algorithm, aimed at stabilizing the retrieval against ongoing degradation of the sensor. The resulting custom SIF dataset is made available along with this publication. Note that gathered spectra from South America are also unavoidably subject to larger measurement errors caused by the increased levels of highly energetic particles in this region, known as the South Atlantic Anomaly. The spatial resolution of the level 3 SIFTER product is 0.5° × 0.5°.

The GOME-2A SIF signals exhibit a negative trend of −1% yr^−1^ due to instrument degradation as carefully documented in Zhang *et al.* [30]. This trend is easily visible in SIF over the Amazon region (figure 2) with larger impacts on the latter part of the record. To remove this trend from the SIF signal and properly account for the 2015/2016 El Niño impact, we applied three different detrending methods over the full period (2007–2016): (1) using a first order polynomial (i.e. linear) to fit the trend, (2) using a second-order polynomial (i.e. quadratic) and (3) using a curve fitting procedure based on Thoning *et al.* [32], also known as CCGCRV. The latter is used widely for time series analysis in the field of atmospheric CO_2_ studies and fits a time series by a combination of a second-order polynomial and four harmonics of different amplitude and phase after filtering the time series for short-term variations, in the frequency domain. A fourth method using principle component analysis was attempted, but found less effective in separating anomalies from the regular seasonal cycle. Method (1) was also tried as 12 separate fits for each calendar month, but this yielded very similar results to (1) suggesting that the negative SIF trend has minimal seasonal differences. Zhang *et al.* [30] came to a similar conclusion for spatial differences in the downward trend.

In this work, we base our figures on the linear detrending from method 1 because it is effective, simple, transparent and easily reproducible for others. In the quantification of the anomalies in the text and tables, we include the range of anomalies based on all three detrending methods, with their differences in estimated GPP reductions not exceeding 0.1 PgC over the periods analysed. Note that despite the detrending of the SIF signal over the Amazon region, the SIF from mid-2016 onwards shows a more rapid decline that persists in 2017 (not shown). We discuss the impact of this in the Discussion and emphasize that our detrending method might not be suitable for every possible application of the SIFTER product.

Additional datasets used in our analysis are described in more detail in the electronic supplementary material. This includes the MPI-BGC GPP product from Beer *et al.* [33], GRACE terrestrial water storage [34] and the precipitation dataset MSWEP [35].

### (b) Spatial analysis

Spatial averaging is based on the mask of the legal Amazon https://doi.org/10.18160/P1HW-0PJ6. This mask was also used in the papers of Gatti *et al.* [9] and van der Laan-Luijkx *et al.* [10] to aggregate results, and ensures consistency between the comparisons. The border of this legal Amazon mask is indicated by the green contour in figure 3.

For the subregions, we rely on Köppen–Geiger (KG)-based definitions of climate zones, which take into account precipitation, temperature and vegetation gradients [36]. This leads to the recognition of three dominant regions within the Amazon basin: Region A (KG-code: Af) with evergreen forest that receive continuously high precipitation (greater than 100 mm per month precipitation); Region B (KG-code: Am) with evergreen forest that is seasonally dry (less than 100 mm per month precipitation) and Region C (KG-code: Aw/As) with a very strong seasonality in precipitation and containing savannah-like vegetation, i.e. the Brazilian ‘Cerrado’. The location of the subregions is shown in the insets in figure 4. Note that these definitions are similar to, but not the same as, those used in Lee *et al.* [26], since they had to focus specifically on small rectangular areas that contained sufficient retrievals. With its much higher coverage, SIFTER attains enough retrievals per 0.5° × 0.5° grid box (electronic supplementary material, figure S1) to allow integration over the climate regions chosen.

The conversion of SIF to GPP is done by fitting a slope and intercept to all annual mean SIFTER/GPP pairs within a region (A, B and C), and applying these fits to all monthly SIF values inside the region. Alternatively, we made the same fits but based on separating points by plant-functional type (tropical, savannah/shrubs, other) and by fitting to the annual mean GPP from SiBCASA [37] instead of from Beer *et al.* [33]. The different slopes and intercepts attained are presented in electronic supplementary material, table S2 and the differences over the three fitting methods are propagated into the range of GPP values quoted in this work. This approach acknowledges that the SIF–GPP relationship can be climate or vegetation dependent [24].

## 3. Results

**Figure 1.**
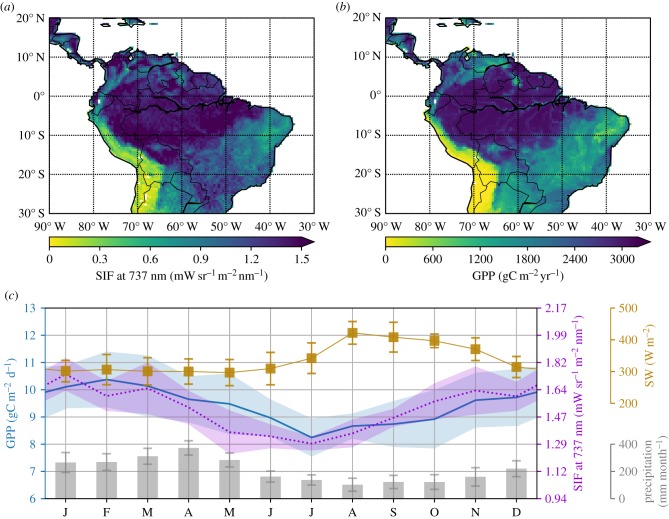
Spatio-temporal distributions of SIFTER fluorescence and observation-based estimates of GPP for the Amazon region and K43 tower. (*a*) Detrended SIFTER signal averaged over 2007–2016 at 0.5° × 0.5° resolution. (*b*) Annual mean MPI-BGC GPP at 0.5° × 0.5° resolution. (*c*) Seasonal cycle of GPP measured at the eddy-covariance tower K34, near Manaus (2.6° S, 60.2° W) averaged over the period 2000–2010. Also shown is the SIFTER product for an aggregated 1.5° × 1.5° cell containing the location of the K34 tower. In addition, the seasonal cycles of the observed precipitation and short wave radiation at the K34 tower are included. The standard deviation of the monthly variables is indicated by either shading or error bars.

The high resolution of our SIF product presents the most detailed view of photosynthetic activity over the Amazon so far. Figure 1 shows the spatial gradients of SIF averaged over 2007–2016 compared to GPP from Beer *et al.* [33], which is partly based on surface NEE observations, partly on remote sensing, and partly on a vegetation-specific relation of GPP to various drivers. The high correlation between GPP and SIF is immediately obvious when looking at the spatial patterns in figure 1. The correlation coefficient for SIF and GPP for all land area shown in figure 1 is *r* = 0.88 (*r* = 0.80 for cells within the Amazon region). Both large-scale gradients across the basin as well as smaller scale gradients such as the forest-savannah transition in central Brazil are captured by SIF. Importantly, we note that in contrast with the GPP product, SIF retrievals have no information on vegetation properties nor distributions, and thus form a fully independent view of the productivity of this region.

**Figure 2.**
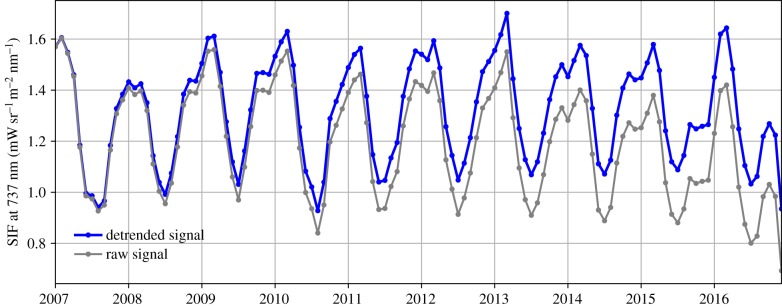
Time series of raw and detrended (see text) SIFTER signals averaged over the legal Amazon region for the period 2007–2016. (Online version in colour.)

The SIFTER product can also capture the seasonal cycle of GPP as measured from the eddy-covariance tower at K34 [38], near Manaus (2.6°S, 60.2°W). Figure 1*c* shows these data averaged over the period 2000–2010, together with the mean seasonal cycle of the detrended SIFTER product for the period 2007–2016 for an aggregated 1.5° × 1.5° cell containing the location of the K34 tower. During JJA, the incoming short wave radiation increases but GPP decreases probably due to a reduced photosynthetic capacity of fresh leaves [39,40]. This is followed by an increase in GPP during September–October when leaf photosynthetic capacity has increased again, a feature missed by most biosphere models that only consider light- and temperature limitations on GPP [41]. Both the initial GPP decrease and its increase in the late dry season at K34 are well captured by the remotely sensed SIFTER product (correlation coefficient *r* = 0.81). The good spatial and temporal correspondence between SIFTER and GPP for the Amazon basin, as demonstrated in figure 1, further motivates the use of SIFTER for analysing the impact of the 2015/2016 El Niño event on the carbon uptake by the Amazon rainforest.

**Figure 3.**
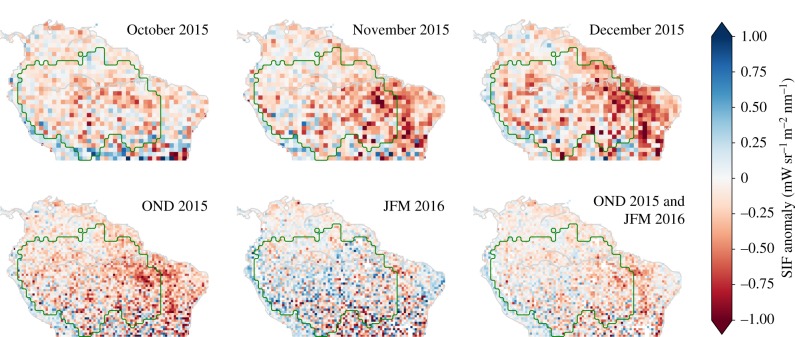
Monthly mean SIF anomalies relative to the climatology for the individual months October–November–December 2015 (top row), as well as for three- and six-month averages (bottom row). Negative values refer to reduced SIF. The resolution of the SIFTER product is 0.5° × 0.5° as shown in the bottom row and each grid cell typically contains between 5 and 12 retrievals (electronic supplementary material, figure S1). The top row has grid cells averaged to 1° × 1° to reduce noise in the monthly spatial patterns. The green contour illustrates the legal Amazon area used throughout this work.

Figure 2 shows the temporal evolution from 2007 through mid-2016 of SIF from the legal Amazon (its extent is indicated in figure 3), which follows a substantial seasonal cycle that ranges over nearly 40% of the long-term average value (1.2 mW sr^−1^ m^−2^ nm^−1^). SIF maxima occur during the wet season and minima during the early dry season seen also by Lee *et al.* [26] and Restrepo-Coupe *et al.* [39]. The long-term decrease of the raw SIF signal starts around 2013 and shows a negative trend that is caused by the functioning of the instrument and the processing of the data. We refer to the electronic supplementary material for an overview of its possible causes. A sharp decline of SIF is visible too at the end of the record (2016–06) and persists into 2017 (not shown), which renders the analysis of post-2016 impossible for now. Despite this, the climatological seasonal cycle, the anomalous dry season SIF in JJA of 2010, and the anomalous early wet season SIF in OND of 2015 are distinguishable, and we attribute this to actually reduced SIF by vegetation. Here, we analyse the latter anomaly further starting from a whole-basin perspective, and then zooming in on regional differences.

Precipitation, SIF values and terrestrial water storage became anomalously low in the Amazon basin (outside the 1−σ range) relative to their climatological seasonal cycle in September, October and prior to December of 2015, respectively (electronic supplementary material, figure S3). The time lag of terrestrial water storage compared to precipitation is expected, and confirmed by detailed simulations of soil moisture anomalies that became anomalously low in October 2015 [37]. The onset of the SIF anomaly corresponds to the peak temperature anomaly (+1.5°C) reported by Jiménez-Muñoz [42], and electronic supplementary material, figure S3*d* shows how SIF in October 2015 remains at the same intensity as the month before, whereas it would normally increase by 10–15% coming out of the dry season. In 2015 though, dry season conditions with below 100 mm per month of precipitation and temperature anomalies persisted much longer (+1°C temperature anomalies continued for at least six months [42]), probably maintaining the water-stress limitations on productivity. By February 2016, basin-integrated SIF had returned to climatological values despite the anomalously low precipitation that persisted throughout the 2016 dry season (electronic supplementary material, figure S3). Terrestrial water storage was last to start recovery (electronic supplementary material, figure S3), which is expected, as it presents the integrated balance between precipitation, run-off and evaporation, which lags precipitation itself. Even in August 2016, it still remained 10% below normal values.

**Figure 4.**
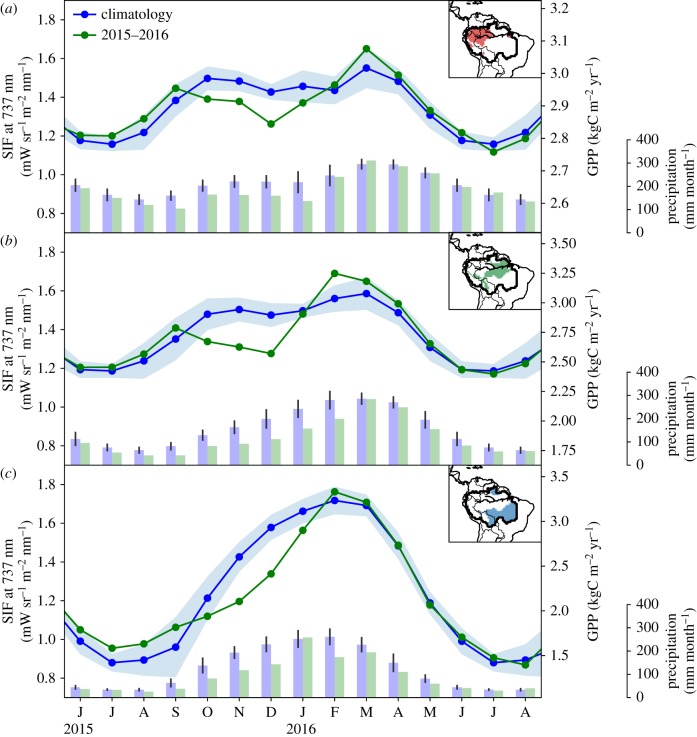
Temporal variation of the climatological SIFTER fluorescence and the 2015/2016 anomalies for different regions inside the legal Amazon based on the Köppen–Geiger climate classification system. The locations of the Regions (A, B, C, see main text for a description) are indicated by the coloured areas on the map insets. Corresponding GPP units are provided on the secondary *y*-axis using region-specific conversion factors (note the different ranges on the GPP-axis). Monthly MSWEP precipitation during the period 2007–2014 (blue) and 2015/2016 (green) are given on the tertiary *y*-axis.

The east-west asymmetry reported by Jiménez-Muñóz [42] for temperature has a strong analogue in SIF (electronic supplementary material, figure S3*a*), with values in the eastern part of the basin more strongly reduced (10–15%) than in the western part (2–5%). In addition to temperature and SIF, this pattern is also present in GRACE water storage (electronic supplementary material, figure S3*b* and Gloor *et al*. [43]), in atmospheric demand for water vapour (electronic supplementary material, figure S4), and in soil moisture anomalies and GPP [37]. We note though that this asymmetry is much less obvious in precipitation anomalies (electronic supplementary material, figure S3*c*), highlighting the role of land–surface interactions in shaping the vegetation response. To account for the climatic variations and different vegetation responses within the Amazon basin, we will focus our further analysis on three distinct subregions (shown in the insets of figure 4), each described in §2b.

Figure 4 shows the climatological seasonal cycle of SIF as well as the 2015/2016 SIF anomalies in these regions. We note that due to the excellent coverage of the SIFTER product, we were able to construct very clear seasonal patterns with a well-defined range of interannual variability, from which the 2015/2016 values clearly deviate beyond the 1−σ standard deviation. The amplitude of the SIF seasonal cycle is largest in Region C, where seasonal rainfall is also most pronounced and drops below 100 mm per month for five months per year. This is in clear contrast with Regions A and B, which have a higher SIF minimum during July and August, but also a lower SIF maximum that is reached first in October–November, and peaks again at the end of the wet season in February–March, when Region C also has maximum SIF.

The 2015/2016 El Niño changed the seasonal cycle in each of the three regions, with the largest relative SIF reductions in Region C. Region C includes both rainforest and savannah, which responds strongly to precipitation [44]. Using a set of linear relations between regional SIF and GPP from the Beer *et al.* [33] product (electronic supplementary material, figure S2) the impact on GPP integrates to the largest reduction in Region C (0.15–0.26 PgC in OND-2015, table 1). This is 16% of its total GPP, and more than twice as large an anomaly as in Region A (8%, or 0.01–0.11 PgC). Region B falls in between these estimates, and together the amount of ‘missed’ GPP relative to the climatology (GPP = 4.8 PgC in OND-2015) is 0.34–0.48 PgC. Note that over the subsequent January–February–March months, the integrated GPP anomaly is very small and even slightly positive (0.06–0.18 PgC), caused by the return of SIF to normal values in Region C and a slightly higher (but within the regular variability) SIF in Regions A and B in February and March. This return to normal values coincides with the return to normal of atmospheric drought conditions, whereas precipitation and soil moisture levels remained low. This atmospheric control suggested by the SIF recovery is discussed further in the Discussion.

**Table 1.** Anomaly in GPP of the terrestrial biosphere for different climate zones in the legal Amazon. Values are derived using three methods for detrending SIFTER fluorescence, two methods for fitting SIF-versus-GPP relations, against two gross primary productivity products [33,45]. Anomalies are integrated over three-month periods, and regions are defined in the main text. Percentages refer to changes relative to the 2007–2014 baseline climatological values, presented in electronic supplementary material, table S1.

regions	area (km^2^)	ΔC_OND_ (PgC)	%	ΔC_JFM_ (PgC)	%
Amazon	7.05 × 10^6^	−0.34 to −0.48	(−8.5%)	+0.06 to +0.18	(+3.1%)
A	1.96 × 10^6^	−0.01 to −0.11	(−2.5%)	+0.01 to +0.013	(+0.3%)
B	2.11 × 10^6^	−0.13 to −0.18	(−10.0%)	+0.05 to +0.09	(+3.2%)
C	2.54 × 10^6^	−0.15 to −0.26	(−15.9%)	−0.02 to +0.04	(+0.7%)

**Figure 5.**
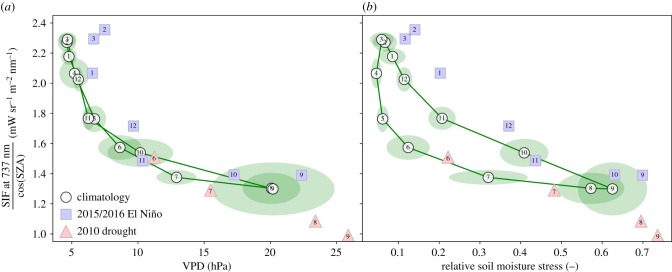
The relationship between (*a*) vapour pressure deficit and (*b*) soil moisture stress, with SIF over Region C of our domain. Green ovals show the variability of VPD or soil moisture stress and SIF in each month of our climatology, labelled in white by the number of the month. Blue numbered squares show the corresponding months during the 2015/2016 El Niño (SOND-JFM), while red numbered triangles are for the 2010 drought event (JJAS). (Online version in colour.)

The strong influence of VPD and soil moisture on SIF is especially clear for Region C. Figure 5 shows the progression of these variables for the climatological seasonal cycle and the concurrent decrease of observed SIF. Note that we have normalized the SIF values using the solar zenith angle to account for the absolute amount of light reaching each point at the satellite overpass time in the Amazon over the seasons. Also, the soil moisture stress is model-derived and could lead or lag the actual climatological plant stress, suggesting a hysteresis in figure 5*b* that is not actually derived from observations.

Highest VPDs of more than 15 hPa occur typically in the dry season in Region C (figure 5), values that are never present over the wetter Regions A and B (electronic supplementary material, figure S5). These values go along with lowest soil moisture values, highest atmospheric water vapour demand (electronic supplementary material, figure S4) and hence highest vegetation stress (with a correlation coefficient of *r* = 0.81). The 2015/2016 El Niño drought occurred in the months following the dry season, and the figure shows how typical values observed for SIF and VPD + soil moisture stress values occurred one month later than normal during El Niño. We note that although both SIF and VPD + soil moisture anomalies are large for the month they occur in, they do not fall outside the range of values experienced by the vegetation during a typical year. This contrasts with the dry season drought of 2010, which peaked in JJA, sending VPD, soil moisture stress and SIF to well outside the regular range. Tentatively, the 2010 drought shows a sign of a change in the VPD–SIF slope at the high end of the VPD range (electronic supplementary material, figures S5 and S6), suggesting that additional drought stress from soil moisture (and possibly also heat) played an important role here too, as discussed below and in [37] (this issue). The return of high SIF values by February and March 2016 in both figures occurs while VPD and soil moisture stress are still somewhat high for the time of year, but at levels that belong to the lowest over a full seasonal cycle. This suggests that the return of clouds and atmospheric moisture, although below typical wet season levels, marked the end of the drought impact on SIF.

## 4. Discussion

Our results demonstrate the substantial impact of the 2015/2016 El Niño on GPP of the Amazon basin, but we caution against a direct extrapolation of the impact on the net carbon balance. Droughts also change the emissions of CO_2_ from fires [10,27,46] and from ecosystem respiration [47,48] and the latter often correlates positively to GPP in its anomaly [12], dampening the impact on net biome exchange (NBE). Several publications that assessed the impact of the 2010 Amazon drought indeed calculated a smaller reduction of net carbon uptake by the vegetation (NBE, 0.0–0.39 PgC yr^−1^ [9–12,48]) than the independently estimated reduction on GPP (0.3–0.8 PgC yr^−1^, [12,26]) and net primary productivity (NPP, 0.14 PgC yr^−1^ [48]). Recent work by Doughty *et al*. [47] furthermore suggests a shift in carbon allocation that increases carbon use efficiency (NPP/GPP) during large droughts, allowing trees to maintain high primary productivity from other sources (most probably carbohydrate reserves) while closing stomata to reduce water-loss (and increase water-use efficiency). This would mean that a SIF-based GPP anomaly would not directly translate to a carbon balance anomaly in vegetation, and that expected relations between environmental drivers (T, VPD, soil moisture, fPAR) and GPP would change during droughts.

Liu *et al.* [5] estimated a substantially higher GPP anomaly over South America from GOSAT SIF (0.9 ± 0.96 PgC) than we report here for October 2015–March 2016 (0.16–0.39 PgC). However, this estimate from Liu *et al.* [5] was based on the entire year 2015 and integrated over a 44% larger mask that also covers parts to the east of the legal Amazon where SIF anomalies were high (electronic supplementary material, figure S7 for a comparison of the masks). The baseline reference period could also play a role, which is much longer in our study than the 2011 La Niña year used as a baseline in their work. Changing our baseline to be only the year 2011 we would find an increase in the GPP anomaly of 0.00–0.15 PgC over the six months we consider in this work.

The results from Liu *et al.* [5] furthermore suggest that the GPP anomaly translated fully to an NBE anomaly. This was partly based on their assessment from space-based CO that fires were not anomalous during the September–March period over which El Niño developed, but also by the need to close the atmospheric CO_2_ mass-balance observed from satellite X_*CO*_2__ column retrievals. To close this balance, a total 0.9 PgC NBE anomaly was needed, thus leaving no room for a contribution from ecosystem respiration. Gloor *et al.* [43] (this issue) estimated a GPP anomaly of 0.9 PgC but over a shorter time window, and a total NBE anomaly of 0.5 PgC, which is also closer to the NBE anomaly of [37] derived from a soil moisture constrained biosphere model. The largest difference between the GPP anomaly estimates from these different sources though is the timing of the recovery of GPP: our SIF product places this three to four months earlier in 2016 than the end of the drought viewed from the perspective of precipitation and soil moisture, and also one to two months earlier than GOSAT SIF and the biosphere model. Further comparisons should thus focus on the period February–March–April 2016 to understand the drought dynamics at the end of the wet season, following the peak anomaly.

According to [42], the 2015/2016 El Niño stands out ‘by having the most extensive area under extreme drought severity (scPDSI less than −4), with up to 13% of the rainforests undergoing extreme drought in February–March 2016’. During this drought, the normally moderately wet Region B received even less precipitation than the seasonally dry Region C, which along with high vapour pressure deficits led to a large soil moisture anomaly [37]. Nevertheless, our SIF product suggests that photosynthesis showed an initial response to the El Niño drought in late 2015 but, with the belated onset of wet season precipitation, SIF returned to (above) normal at the end of the wet season (February–March). Especially in Region B, most drought indicators suggest that anomalous environmental conditions persisted into the 2016 dry season (June–July) before returning to normal. But atmospheric demand for water vapour (i.e. potential evapotranspiration) returned to normal much earlier, and in our analysis seems to have ended drought stress on vegetation leading to a recovery of SIF/GPP. With our SIF retrievals becoming less reliable throughout 2016 though, it is difficult to say whether (a) the recovery was only temporary as fresh rain brought some relief, but SIF and GPP declined strongly again during the 2016 dry season, or (b) the recovery persisted and SIF remained near climatological values for most of 2016. The recovery of SIF we find in February–March is robust against detrending in our analysis though and provides a strong indication that the end of the SIF/GPP response of the 2015/2016 El Niño was under atmospheric control.

Other factors can play a role in comparing our results to existing estimates. Our SIF product only covers scenes with moderate to no cloud cover (cloud fraction less than 0.4), and we thus typically see the part of the canopy that receives a lot of direct sunlight. This means that, through SIF, we would not see the GPP response of the fraction of vegetation that is more strongly shaded by clouds. This would be the less productive part of the forest under non-stressed conditions, but possibly a more productive area under drought stressed conditions. Since large-scale cloud cover also changes during El Niño we have verified (electronic supplementary material, figure S1) that our cloud selection does not influence the retrieval coverage across the different years. We furthermore note that GOME-2 and GOSAT have local overpass times of 9.30 and 13.00, respectively, and that VPD and stomatal closure will change during the day. In a model-based analysis, this changed the GPP/SIF correlation and slope, with smaller VPD impact on GPP in the morning. This could complicate a comparison between the different SIF products but was found to have no effect on the GOME-2A anomalies presented in this paper. Similar to GOSAT, the recently launched TROPOMI instrument provides a view on the more drought-affected afternoon. However, TROPOMI attains daily global coverage with a high spatial resolution of 7 × 7 km, and is therefore a promising tool for future drought studies, especially over clouded tropical rainforests [22].

## 5. Conclusion

We have presented an analysis of spatio-temporal patterns of SIF across the Amazon basin using a new retrieval product that reduces noise and improves signals particularly for tropical regions, as evidenced by high correlations with the observation-driven Beer *et al.* [33] GPP product and independent flux-tower data. Our results show a clear difference in SIF response to droughts from the western to the eastern part of the legal Amazon basin, which was not detectable in the gradients from GOME-2 presented before [28], nor discussed in the earlier GOSAT SIF-based study by Liu *et al.* [5]. This pattern is in very good agreement with the locations of the largest anomalies of temperature [42], evapotranspiration, terrestrial water storage [28,43] and soil moisture [37]. Anomalies in all these variables start to build up after September 2015 and accumulate to a 0.51 PgC GPP anomaly by the end of January. The largest contribution to this anomaly comes from the seasonally dry vegetation in the south-eastern part of the basin, with smaller contributions from the wetter regions in the northeast where the drought is most intense. By February 2016, SIF returns to climatological values in our product despite persisting anomalies in temperature, precipitation, terrestrial water storage and soil moisture into the following January 2016 dry season. This suggests that the return to wet season conditions was sufficient to rapidly bring SIF back to normal levels, ending the drought from a GPP perspective.

## Supplementary Material

Supplemental Information

## Data accessibility

The Amazon dataset used in this paper is based on a customized retrieval that builds on further developments of the SIFTER v2 algorithm. The customized dataset is available through its DOI https://doi.org/10.18160/ECK0-1Y4C. A global prototype SIFTER v2 dataset and a preliminary Algorithm Theoretical Baseline Document [49] are available through the data portal at http://www.temis.nl.
