# Supplementary material for: Widespread reduction in sun-induced fluorescence from the Amazon during the 2015/2016 El Niño
Source: Philos Trans R Soc Lond B Biol Sci. 2018 Oct 8;373(1760):20170408. doi: 10.1098/rstb.2017.0408 (PMC6178432; doi:10.1098/rstb.2017.0408)
Supplement: Supplemental Information [file rstb20170408supp1.pdf]

Supplemental Information for "Widespread reduction in sun-induced fluorescence from the Amazon during the 2015/2016 El Niño"

Gerbrand Koren[a], Erik van Schaik[a], Alessandro C. Araújo[b], K. Folkert Boersma[a,c], Antje Gärtner[a], Lars Killaars[d], Maurits L. Kooreman[c], Bart Kruijt[a], Ingrid T. van der Laan-Luijkx[a], Celso von Randow[e], Naomi E. Smith[a], Wouter Peters[a,d]

[a] *Wageningen University and Research, Wageningen, The Netherlands*
[b] *Embrapa Amazônia Oriental CPATU, Belem, Brazil*
[c] *Royal Netherlands Meteorological Institute (KNMI), De Bilt, The Netherlands*
[d] *University of Groningen, Centre for Isotope Research, Groningen, The Netherlands*
[e] *Instituto Nacional de Pesquisas Espaciais, São José dos Campos, Brazil*

Contents

- Sections S1 – S5
- Figures S1 – S7
- Tables S1 and S2
- References

S1. SIFTER fluorescence

Sun-induced fluorescence (SIF) is the re-emission of light by the chloroplast during photosynthesis. Since SIF is directly linked to the photosynthesis process, it can serve as a proxy for photosynthetic CO_2 uptake by vegetation. It was shown in laboratory and field studies that SIF can be used to detect water stress of vegetation [1, 2]. SIF can also be measured from space, which provides a unique opportunity to study large-scale vegetation response to climatic events.

We use SIF obtained from the Global Ozone Monitoring Experiment-2 (GOME-2) instruments on board of the MetOp-A satellite that is operated by the European Organization for the Exploitation of Meteorological Satellites (EUMETSAT). The MetOp-A satellite was launched in October 2006, followed by the MetOp-B satellite in September 2012. MetOp-C, a third satellite in this series, is scheduled to be launched in 2018. The MetOp satellites have a sun-synchronous orbit and have a local overpass time of 9:30 in the morning.

Our analysis is based on a custom version of Sun-Induced Fluorescence of Terrestrial Ecosystems Retrieval (SIFTER) developed at the Royal Netherlands Meteorological Institute (KNMI). This SIFTER version is an update of the retrieval described by Sanders et al. [3]. The SIFTER retrieval is based on a stochastic representation of the atmospheric reflectance spectrum by a set of principal components. These principal components are derived from GOME-2 observations over a reference region over the Sahara where vegetation (and hence SIF) is absent. In the retrieval algorithm the surface albedo and fluorescence are estimated from the GOME-2 observations and the principal components by minimizing a cost function. Note that SIF is retrieved without ancillary datasets such as pre-defined land cover type maps.

The following major changes were made in the retrieval method for SIFTER: (1) the principal components representing the atmospheric disturbance are now determined for the period from 2007-2016, instead of a moving yearly window; (2) the spectral fitting window is reduced from 712-783 nm to 734-758 nm and the number of principal components used to simulate atmospheric effects is reduced from 35 to 8; (3) SIF retrievals are rejected when autocorrelation in the fit residuals is larger than 0.2; and (4) a correction for the remaining irradiance bias and latitudinal bias was applied.

SIFTER values were retrieved with a set of principal components that represents the atmospheric effects on measured irradiance. As mentioned

above, the principal components are derived from the period 2007-2016 for a reference area over the Sahara. Experiments with different settings for the retrieval algorithm have shown that the inter-annual variability (IAV) in the SIF signal strongly increases when a shorter reference periods is selected (data not shown). A possible explanation for this effect is that the IAV in the water vapour over the Saharan reference area translates into IAV in the retrieved SIF signal for e.g. the Amazon region.

Analysis of the raw SIF signal reveals that there is a negative trend in the SIF signal over the Amazon region (see Figure 2). To remove the trend from the SIF signal, we used the following detrending methods: (1) linear detrending; (2) quadratic detrending; and (3) detrending using the CCGCRV routine that was developed by Thoning et al. [4] for the analysis of CO_2 time series. The effect of the selected detrending method on the resulting GPP anomalies is discussed in the main text.

Possible explanations for the observed SIF trend are changes related to (1) the physical instruments (e.g. sensor degradation or drift of the satellite from its original orbit); (2) the instrument settings and low level data processing (e.g. the pixel size that changed for GOME-2A from 80×40 km^2 to 40×40 km^2 in 2013 and changes in the processing of level 0 and level 1 data) or (3) geophysical changes in the Earth system (e.g. the atmospheric composition which can make the set of principal components less representative). Our method, using a long baseline period to derive these PCs and reduce noise, is likely more sensitive to this decline in signal than other GOME retrievals.

S2. Coverage of SIFTER over time

Figure S1 shows the mean number of 'valid' samples that were used to determine the monthly mean SIF signal for the 0.5°×0.5° grid boxes within the different subregions of the legal Amazon. The most important reasons for disregarding an observation are (1) cloud fraction over the grid box is higher than the maximum of 0.4; (2) the autocorrelation in the residue is higher than 0.2, which is an indication of an erroneous fit [5]. Note that each subregion A, B, or C contains 636, 688, and 840 gridboxes respectively, such that the total number of SIF values used per aggregated region is highly robust.

The number of samples per month follows a strong seasonal cycle and also an upward trend, similar to the downward trend that we find in the raw SIFTER signal. As noted above, the number of samples per grid box

per month contains indirect information about the clouds, since a maximum cloud fraction of 0.4 was imposed. The seasonal cycle in number of retrievals thus closely follows the seasonal cycle of cloudiness, and thus the wet seasons and dry seasons.

The number of samples over the Amazon regions is about 50% lower than the number of samples over all tropical land (from 30°N-30°S, including the Amazon region itself, data not shown), which is due to (1) the above average cloudy conditions over the forested Amazon and (2) the noise originating from the South Atlantic Anomaly.

S3. Additional datasets

S3.1. MPI-BGC gross primary productivity

We use the annual mean GPP product from the Max Planck Institute for Biogeochemistry (MPI-BGC) [6] that was created by extrapolating a database of eddy-covariance measurements using different machine learning approaches. This GPP product has a spatial resolution of 0.5°×0.5°.

S3.2. GRACE terrestrial water storage

We use the GRACE JPL-RL05M mascon product from the Jet Propulsion Laboratory (JPL). This mascon product is based on prior model information which results in a better signal-to-noise ratio than GRACE products based on the spherical harmonics retrieval [7].

The JPL-RL05M GRACE data is available from April 2002 to June 2017 at (nearly) monthly time resolution and a spatial resolution of 3°×3°. This dataset is hosted in the data portal at `https://podaac.jpl.nasa.gov/` [8].

Note that the JPL GRACE product does not represent an absolute value for the terrestrial water storage, but it is a series of anomalies in terrestrial water storage. Since these anomalies are defined relative to a multi-year baseline (2004-2009), the anomalies still contain a seasonal cycle.

S3.3. MSWEP precipitation

We also use precipitation from the Multi-Source Weighted-Ensemble Precipitation (MSWEP) [9] dataset in this study. MSWEP is a global precipitation dataset derived from gauge measurements, satellite observations and re-analysis products. The spatial resolution of MSWEP is 0.1°×0.1° and MSWEP has a 3-hourly temporal resolution. It was found in a comparative

study that MSWEP has the highest correlation with local gauge measurements out of 22 precipitation products [10]. The MSWEP product is available through the data portal `http://www.gloh2o.org/`.

S4. SIF-GPP scaling

To quantify the observed SIF-reductions in terms of PgC, a conversion from SIF to GPP is required. We derive a linear fit for GPP vs. SIF, similar to Parazoo et al. [11]. Figure S2 shows GPP from Beer et al. [6] versus annual mean SIFTER for cells within the Amazon region. To prevent that large concentrations of points within a small SIF range are dominating the constructed fit, we have first calculated the mean GPP over equal bins of 0.2 $mW{\cdot}sr^{-1}{\cdot}m^{-2}{\cdot}nm^{-1}$. Subsequently, the fit was determined based on the binned representation of the data, where equal weights were assigned to each bin. Bins with less than 3 points were discarded.

The default set-up for calculating the GPP-SIF slope is using a linear SIF detrending, for different climate zones (Regions A, B and C) and using the Beer et al. [6] GPP product (see Figure S2). For our error analysis we also use the quadratic and CCGCRV [4] detrending method as alternative to the linear detrending. Furthermore we tested the effect of plant functional type (PFT) specific scaling as alternative to the division in climate zones. Finally, we used GPP from terrestrial biosphere model SiBCASA [12] as alternative to the Beer et al. [6] GPP product. The resulting slopes and intercepts are summarized in Table S2.

S5. SIF correlations with environmental variables

Figure S5 shows the relationship between SIF and VPD after aggregation over the regions A, B, and C (see main text for definition), for each month of our climatology (colour) as well as for the 2015/2016 El Niño and the 2010 drought event. It illustrates the larger sensitivity of regions B, and especially C, to VPD compared to Region A. Highest VPD values are consistently found over Region C. Lowest normalized SIF values correspond to these points, with the 2010 drought having the highest impact and the relation appearing to drop below the exponential curve that would connect the coloured (climatological) values.

Figure S6 presents this same view, but now using soil moisture stress on the horizontal axis. This is derived from the simulations presented in van

Schaik et al. [13] (this issue). High values of relative soil moisture stress correspond to high reductions in SIF. The figure shows that VPD and soil moisture stress likely both play a role in reducing SIF over Region C, but likely also have contributed to the reduction of SIF in Region B. Disentangling this relative influence is challenging, but would give further insights into the drought dynamics of vegetation in this region.

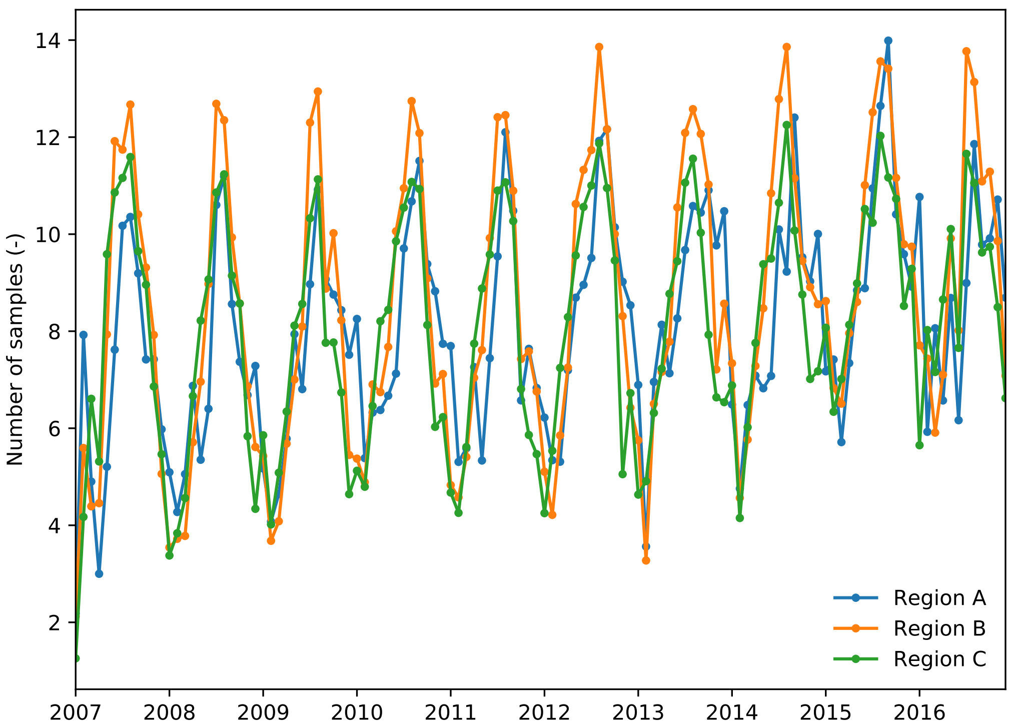

Figure S1: Time series of the mean number of samples constituting the monthly SIF from SIFTER retrieval of GOME-2A for different regions inside the legal Amazon.

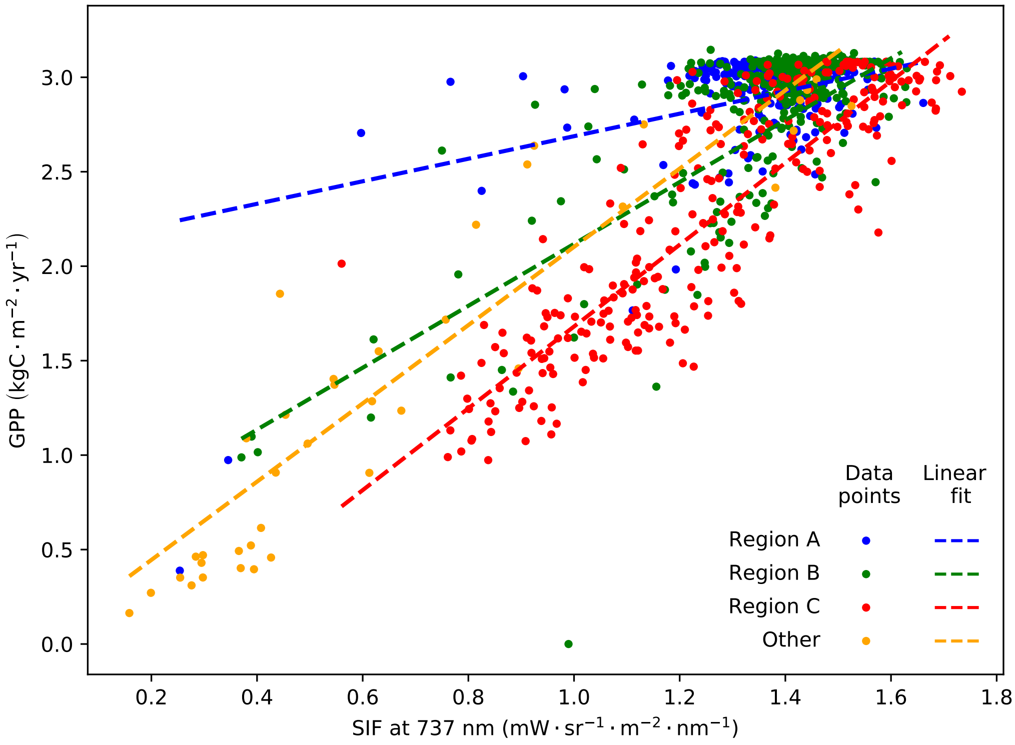

Figure S2: GPP from the Beer et al. [6] product versus annual mean detrended SIFTER fluorescence for each 0.5°×0.5° grid box within the Amazon region. Colours correspond to the climate zones defined in the main text.

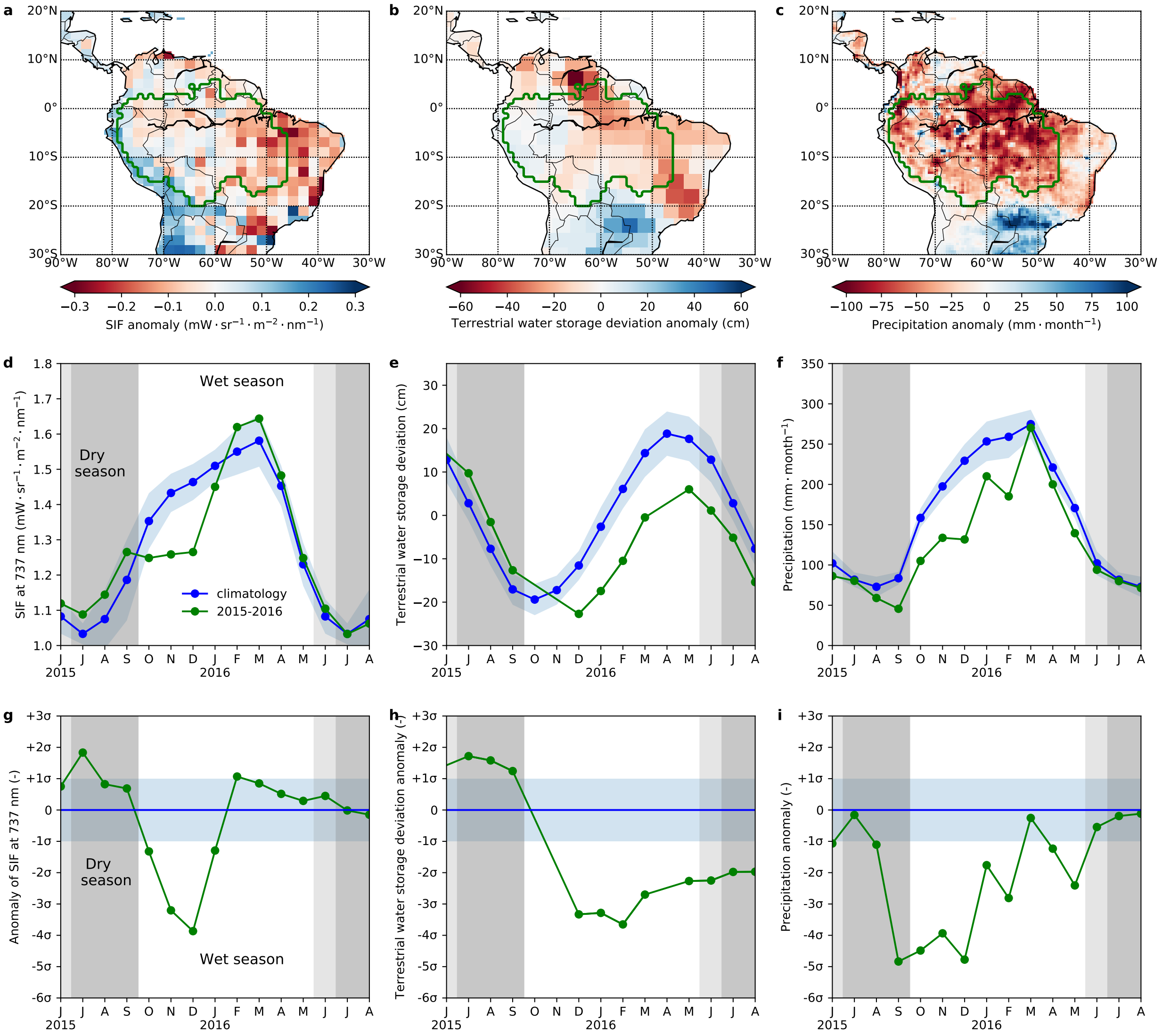

Figure S3: Spatio-temporal distributions of 2015/2016 anomalies in the Amazon region of **(a, d, g)** SIFTER fluorescence; **(b, e, h)** GRACE terrestrial water storage anomalies; and **(c, f, i)** MSWEP precipitation. The spatial distributions show the cumulative anomaly of the variable integrated over the period September 2015 to May 2016 for the northern part of South America. The location of the legal Amazon region is indicated by a thick green line. The time series show the climatological values, and values during the 2015/2016 El Niño period integrated over the legal Amazon region. The bands surrounding the climatological values indicate the year-to-year variability (1-σ) around the climatology. For SIF and precipitation we used the period 2007-2014, whereas the baseline for GRACE terrestrial water storage is 2002-2014. The dark grey shaded area indicates the climatological dry season (< 100 mm/month precipitation) for the Amazon region, the light grey shaded area indicates the extended dry season for 2015 and 2016.

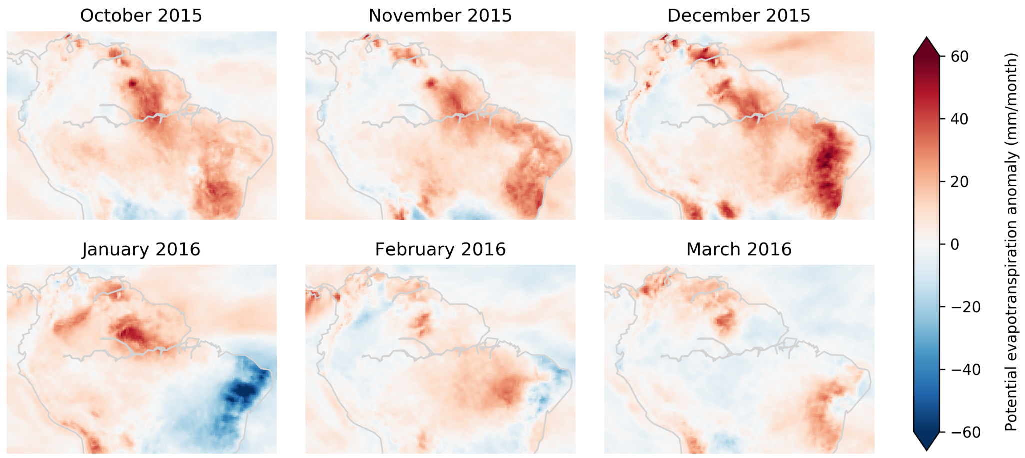

Figure S4: Anomaly of potential evapotranspiration, relative to the 2010-2016 period. Red colours show higher than normal atmospheric demand. Numbers are based on a Penman-Monteith calculation with ERA5 atmospheric variables.

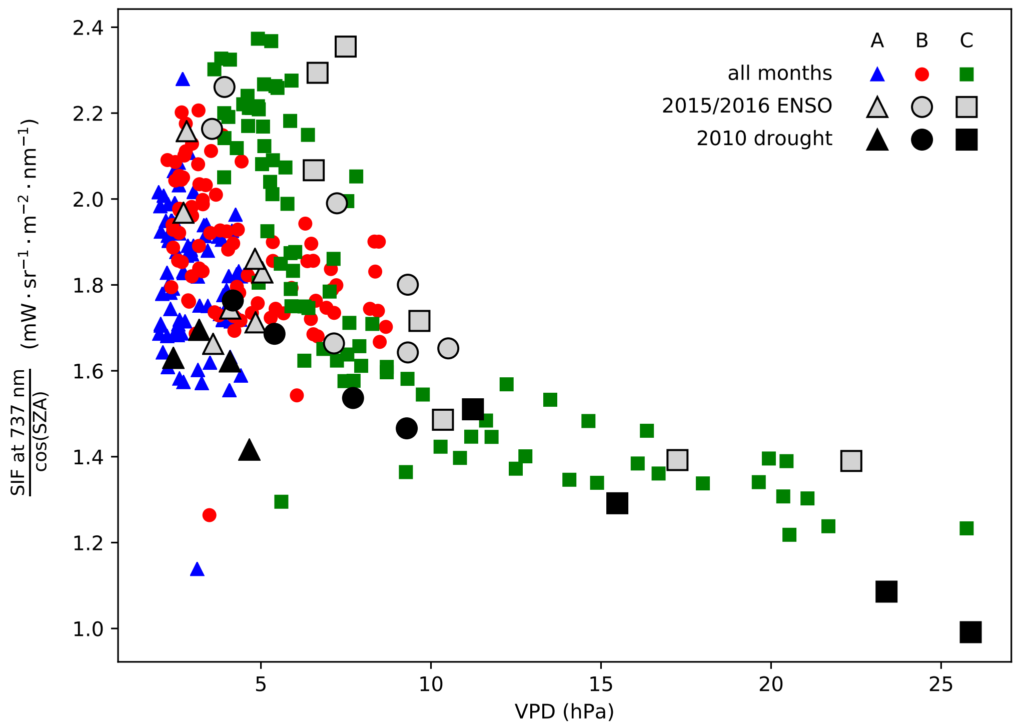

Figure S5: Average SIF for each region normalised by the cosine of the solar zenith angle, versus VPD. Each dot corresponds to one month from either the climatology (colours), from the 2015/2016 El Niño period (gray), or from the 2010 drought (black). Shapes and colours are used to distinguish between Regions A, B, and C. A summary view of this figure is presented in the main text.

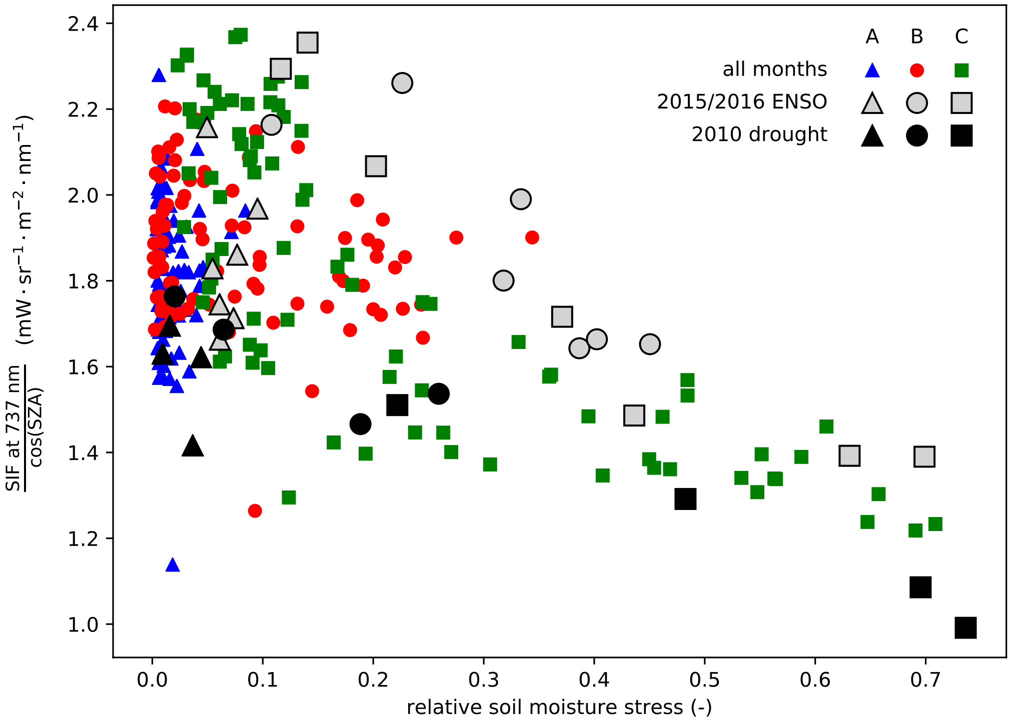

Figure S6: Average SIF for each region normalised by the cosine of the solar zenith angle, versus relative soil moisture stress as calculated by van Schaik et al. [13]. Each dot corresponds to one month from either the climatology (colours), from the 2015/2016 El Niño period (gray), or from the 2010 drought (black). Shapes and colours are used to distinguish between Regions A, B, and C. A summary view of this figure is presented in the main text.

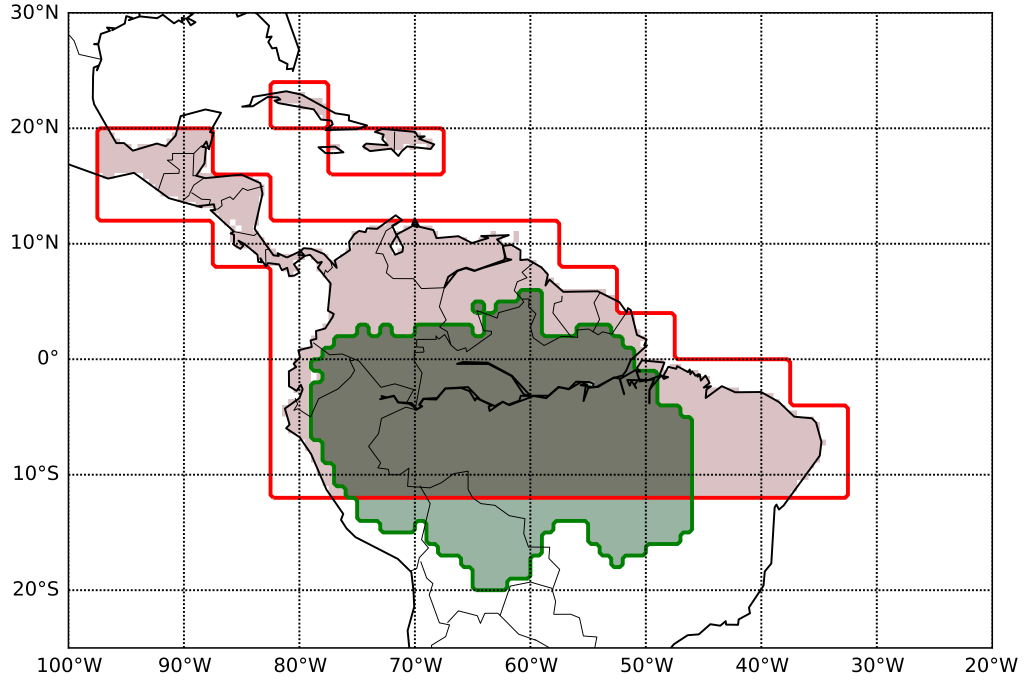

Figure S7: Comparison of the Legal Amazon mask (`https://doi.org/10.18160/P1HW-0PJ6`) used in this study and the "Trop SA" mask used by Liu et al. [14]. The Liu et al. [14] mask is about 44% larger in land area and contains 34% more GPP according to the Beer et al. [6] GPP product.

Table S1: Climatological GPP for different climate zones in the Amazon regions. Values are derived from SIFTER fluorescence and MPI-BGC gross primary productivity (Beer et al. [6]), and integrated over 3-month periods, and regions defined in Sect 2.2 of the main text.

Regions	Area (km^2)	SIF ($mW \cdot sr^{-1} \cdot m^{-2} \cdot nm^{-1}$)		GPP (PgC)	
		OND	JFM	OND	JFM
Legal Amazon	$7.05 \cdot 10^6$	1.42	1.55	4.83	5.29
Region A	$1.96 \cdot 10^6$	1.47	1.48	1.45	1.46
Region B	$2.11 \cdot 10^6$	1.49	1.55	1.54	1.59
Region C	$2.54 \cdot 10^6$	1.41	1.69	1.63	2.02
Other	$4.34 \cdot 10^5$	0.90	0.99	0.21	0.23

Table S2: Values of the GPP/SIF slope derived for the Amazon.

**Aggregation**	**Slope** [kgC·yr^{-1}/ mW·sr^{-1}·nm^{-1}]	**Intercept** [kgC·m^{-2}·yr^{-1}]	**SIF range** [mW·sr^{-1}·m^{-2}·nm^{-1}]
	Climate zones, Amazon, GOME-2A vs Beer et al. [6]		
Region A	0.60	2.09	0.8–1.6
Region B	1.64	0.48	0.6–1.8
Region C	2.17	-0.48	0.6–1.8
Other	2.07	0.03	0.2–1.6
All Amazon	1.80	0.18	0.2–1.8
	PFTs, Amazon, GOME-2A vs Beer et al. [6]		
Tropical forest	1.38	0.84	0.6–1.8
Savanna	1.78	0.08	0.2–1.8
Others	1.99	-0.14	0.2–1.8
	Climate zones, Amazon, GOME-2A vs SiBCASA [12]		
Region A	1.87	0.85	0.8–1.6
Region B	1.86	0.32	0.6–1.8
Region C	1.41	0.30	0.6–1.8
Other	3.35	-1.56	0.2–1.6
All Amazon	2.31	-0.53	0.2–1.8
	Parazoo et al. [11], global, GOSAT vs Beer et al. [6]		
Evergreen broad-leaf forest	5.77	0.62	0.05–0.45
Savanna	5.59	0.22	0.05–0.38
All	6.63	0.04	-0.1–0.45

References

[1] J. Flexas, J. M. Escalona, S. Evain, J. Gulias, I. Moya, C. B. Osmond, H. Medrano, Steady-state chlorophyll fluorescence (Fs) measurements as a tool to follow variations of net CO_2 assimilation and stomatal conductance during water-stress in C_3 plants, Physiologia Plantarum 114 (2002) 231–240.

[2] F. Daumard, S. Champagne, A. Fournier, Y. Goulas, A. Ounis, J.-F. Hanocq, I. Moya, A field platform for continuous measurement of canopy fluorescence, IEEE Transactions on Geoscience and Remote Sensing 48 (2010) 3358–3368.

[3] A. Sanders, W. Verstraeten, M. Kooreman, T. van Leth, J. Beringer, J. Joiner, Spaceborne sun-induced vegetation fluorescence time series from 2007 to 2015 evaluated with Australian flux tower measurements, Remote Sensing 8 (2016) 895.

[4] K. W. Thoning, P. P. Tans, W. D. Komhyr, Atmospheric carbon dioxide at Mauna Loa Observatory: 2. Analysis of the NOAA GMCC data, 1974-1985, Journal of Geophysical Research: Atmospheres 94 (1989) 8549–8565.

[5] E. van Schaik, Retrieving Sun-Induced Fluorescence from the Global Ozone Monitoring Experiment-2, Wageningen University MSc Internship report for Royal Netherlands Meteorological Institute (KNMI), 2016. Available at https://goo.gl/fgAMG7.

[6] C. Beer, M. Reichstein, E. Tomelleri, P. Ciais, M. Jung, N. Carvalhais, C. Rodenbeck, M. A. Arain, D. Baldocchi, G. B. Bonan, A. Bondeau, A. Cescatti, G. Lasslop, A. Lindroth, M. Lomas, S. Luyssaert, H. Margolis, K. W. Oleson, O. Roupsard, E. Veenendaal, N. Viovy, C. Williams, F. I. Woodward, D. Papale, Terrestrial gross carbon dioxide uptake: global distribution and covariation with climate, Science 329 (2010) 834–838.

[7] M. M. Watkins, D. N. Wiese, D.-N. Yuan, C. Boening, F. W. Landerer, Improved methods for observing Earth's time variable mass distribution with GRACE using spherical cap mascons, Journal of Geophysical Research: Solid Earth 120 (2015) 2648–2671.

[8] D. N. Wiese, D.-N. Yuan, C. Boening, F. W. Landerer, M. M. Watkins, JPL GRACE mascon ocean, ice, and hydrology equivalent water height RL05M.1 CRI filtered version 2, 2016.

[9] H. E. Beck, A. I. J. M. van Dijk, V. Levizzani, J. Schellekens, D. G. Miralles, B. Martens, A. de Roo, MSWEP: 3-hourly 0.25° global gridded precipitation (1979-2015) by merging gauge, satellite, and reanalysis data, Hydrology and Earth System Sciences 21 (2017) 589–615.

[10] H. E. Beck, N. Vergopolan, M. Pan, V. Levizzani, A. I. J. M. van Dijk, G. P. Weedon, L. Brocca, F. Pappenberger, G. J. Huffman, E. F. Wood, Global-scale evaluation of 22 precipitation datasets using gauge observations and hydrological modeling, Hydrology and Earth System Sciences 21 (2017) 6201–6217.

[11] N. C. Parazoo, K. Bowman, J. B. Fisher, F. Christian, D. B. A. Jones, A. Cescatti, . Pérez-Priego, G. Wohlfahrt, L. Montagnani, Terrestrial gross primary production inferred from satellite fluorescence and vegetation models, Global Change Biology 20 (2014) 3103–3121.

[12] K. Schaefer, G. J. Collatz, P. Tans, A. S. Denning, I. Baker, J. Berry, L. Prihodko, N. Suits, A. Philpott, Combined Simple Biosphere/Carnegie-Ames-Stanford Approach terrestrial carbon cycle model, Journal of Geophysical Research 113 (2008) G03034.

[13] E. van Schaik, L. Killaars, N. E. Smith, G. Koren, L. P. H. van Beek, W. Peters, I. T. van der Laan-Luijkx, Changes in surface hydrology, soil moisture, and Gross Primary Productivity in the Amazon during the 2015/2016 El Niño, Philosophical Transactions of the Royal Society B (2018).

[14] J. Liu, K. W. Bowman, D. S. Schimel, N. C. Parazoo, Z. Jiang, M. Lee, A. A. Bloom, D. Wunch, C. Frankenberg, Y. Sun, C. W. O'Dell, K. R. Gurney, D. Menemenlis, M. Gierach, D. Crisp, A. Eldering, Contrasting carbon cycle responses of the tropical continents to the 2015-2016 El Niño, Science 358 (2017) eaam5690.
